# Surface nanogrooving of carbon microtubes

**DOI:** 10.1038/s41598-018-28313-0

**Published:** 2018-07-02

**Authors:** Bijan Nasri-Nasrabadi, Akif Kaynak, Zahra Komeily-Nia, Scott D. Adams, Jingliang Li, Abbas Z. Kouzani

**Affiliations:** 1 School of Engineering, Deakin University, Geelong, Victoria, 3216 Australia; 2 Institute for Frontier Materials, Deakin University, Geelong, Victoria, 3216 Australia

## Abstract

Extrusion processing of carbon tubes can be problematic due to their poor interfacial interactions with polymeric matrices. Surface chemical modification of carbon tubes can be utilized to create bonding sites to form networks with polymer chains. However, chemical reactions resulting in intermolecular primary bonding limit processability of extrudate, since they cause unstable rheological behaviour, and thus decrease the stock holding time, which is determinative in extrusion. This study presents a method for the synthesis of carbon microtubes with physically modified surface area to improve the filler and matrix interfacial interactions. The key concept is the formation of a nanogrooved topography, through acoustic cavitation on the surface of processing fibres. The effect of nanogrooving on roughness parameters is described, along with the role of surface modified carbon tubes on rheological behaviour, homogeneity, and coherency of extrudate. The measurements showed that nanogrooving increases the surface area of carbon microtubes, as a result, die swelling of the extrudate is reduced. Furthermore, after solidification, the mechanical strength of composite is reinforced due to stronger interactions between nanogrooved carbon tubes and polymer matrix.

## Introduction

Carbon tubes are allotropes of carbon with an exceptional combination of stiffness, low density, and electrical conductivity^1^. On the microscopic scale, these properties result in lightweight composite structures, combining the high specific mechanical strength of polymers and electrical conductivity of graphite^2,3^. For the fabrication of carbon tube assemblies, wet spinning is the first choice. In this technique, the carbon tubes are dissolved in either super acids^4–6^ or polymer solutions^7–9^, extruded through a nozzle, and finally coagulate into fibre structures with a high degree of orientation. For the majority of anisotropic building fillers^10,11^, shear rate-induced alignment endows the fabricated structures with enhanced properties. Despite their excellent properties, development of self-supported structures from carbon tubes is challenging due to their poor surface functionality. Chemical modification is considered to be a versatile method for fabricating reinforced composites by facilitating the formation of a thorough bonding network between filler and matrix^12^. However, chemical crosslinking restricts the freedom of polymer chains and therefore considerably changes the rheological behaviour of the composite solution^13,14^, which is critical in extrusion. Nozzle blockages may arise due to the time dependant changes in the viscosity, which are attributed to intermolecular chemical reactions. Additionally, chemical functionalization limits the holding time of the solution before processing^15,16^. Therefore, a physical approach for treating the surface of carbon tubes to achieve enhanced interactions with a polymer matrix is desired. In this work, we created nanogrooves on the surface of carbon tubes with a view to achieve enhanced physical entanglements with the polymer as well as efficient coagulation.

## Results and Discussion

### Effect of pre-treatment

**Figure 1:**
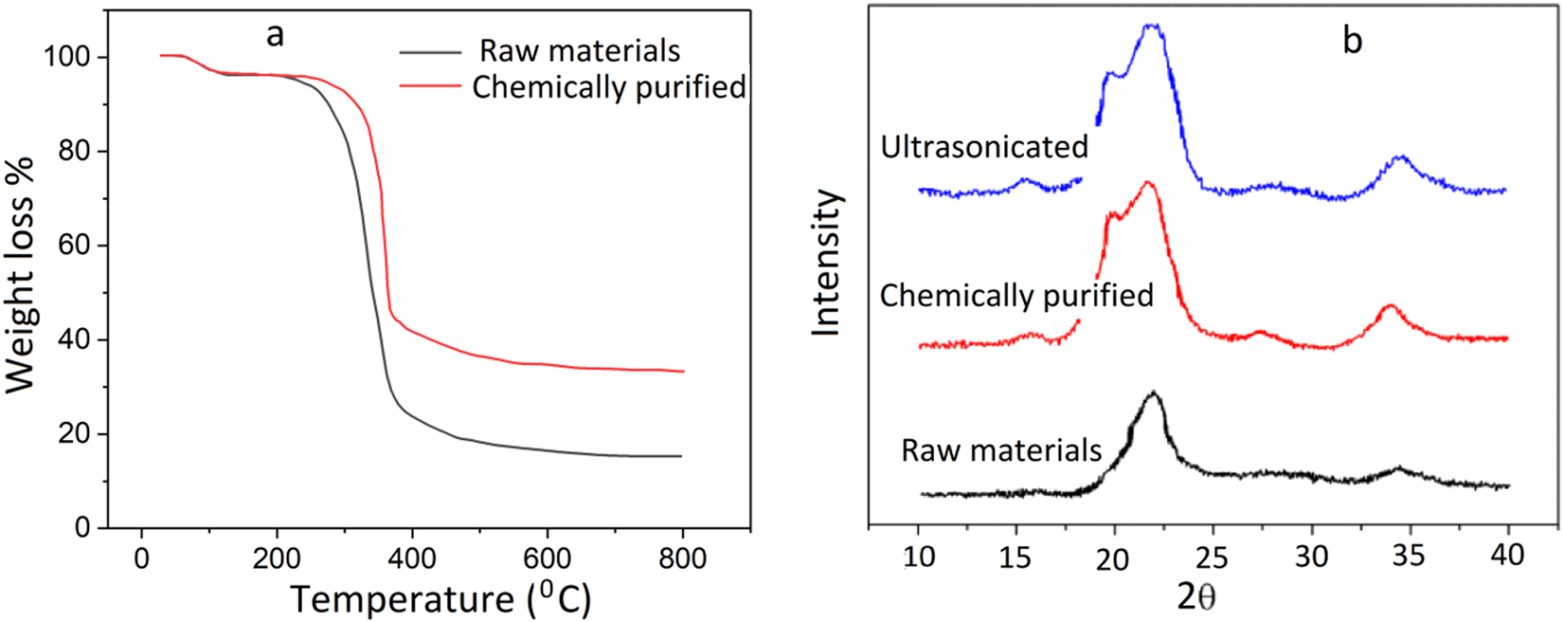
(**a**) TGA curves of raw and chemically purified fibres (**b**) X-ray diffraction patterns of cellulose fibres at different treatment stages.

Thermal gravimetric analysis (TGA) was used to evaluate the effect of chemical treatment on the purification of cellulosic fibres before carbonization. Figure 1a shows the TGA curves of untreated and chemically purified fibres. For both samples a small weight loss was observed at around 100 °C that is related to the evaporation of low molecular weight components and humidity from the fibres. The next main weight loss is related to hemicellulose and cellulose pyrolysis that is shifted from 250 °C for the raw materials to 330 °C for the chemically purified fibres. This shift is due to the removal of hemicellulose and amorphous cellulose after chemical treatment.

Powder X-ray diffraction was used to show the effect of chemical treatment and ultrasonication on crystallinity of fibres. Raw fibres have diffraction peaks (2θ) around 16.5° and 22.5° (with the crystallinity index of 55%) that typically displays the pattern of cellulose type Ι^16,17^ (Fig. 1b). However, chemical purification changed the pattern to cellulose type Π, where the split peak moved to deflection angles (2θ) of approximately 20° and 21.7° with the crystallinity index of 74%. No substantial change was induced by sonication. XRD pattern of cellulose type Π with the crystallinity index of 72% shows that the high tension sonication had little influence on crystalline regions of microfibers. This result was similar to those reported on purified wood cellulose suspension, where ultrasonication did not lead to a significant effect on crystalline structure of fibres^18^.

**Figure 2:**
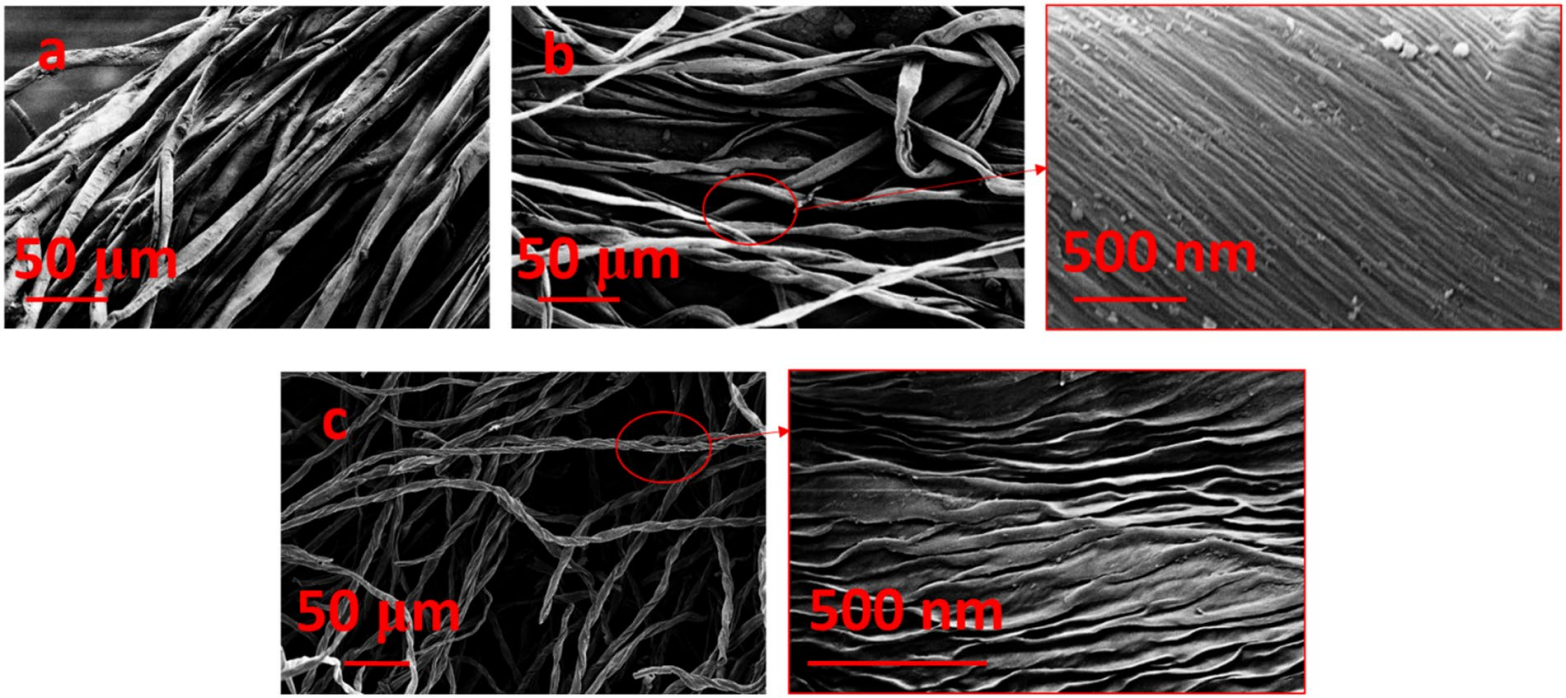
SEM images of (**a**) after lignin leaching with sodium chlorite, (**b**) after purification with potassium hydroxide, and (**c**) after high-tension sonication (400 W).

Figure 2a–c are the SEM images of raw, chemically treated, and ultrasonicated cellulose fibres. Chemical treatment separated the cotton fibres into microfibers. These microfibers are composed of nanofiber bundles, networked by hydrogen bonds^19^. Figure 2b shows the bundles on the surface of the purified fibres in a cohesive network. High tension sonication individualized the nanoscale bundles on the surface of fibres (as can be observed in Fig. 2c). As a consequence of expansion and implosion of microbubbles in the dispersion acoustic cavitation occurs on the microfiber structure. The phenomenon creates shock waves and microjets on the surface of microfibers, causing the hydrogen bonding networks to loosen and nanofibres bundles to split^19,20^.

### Effect of carbonization

**Figure 3:**
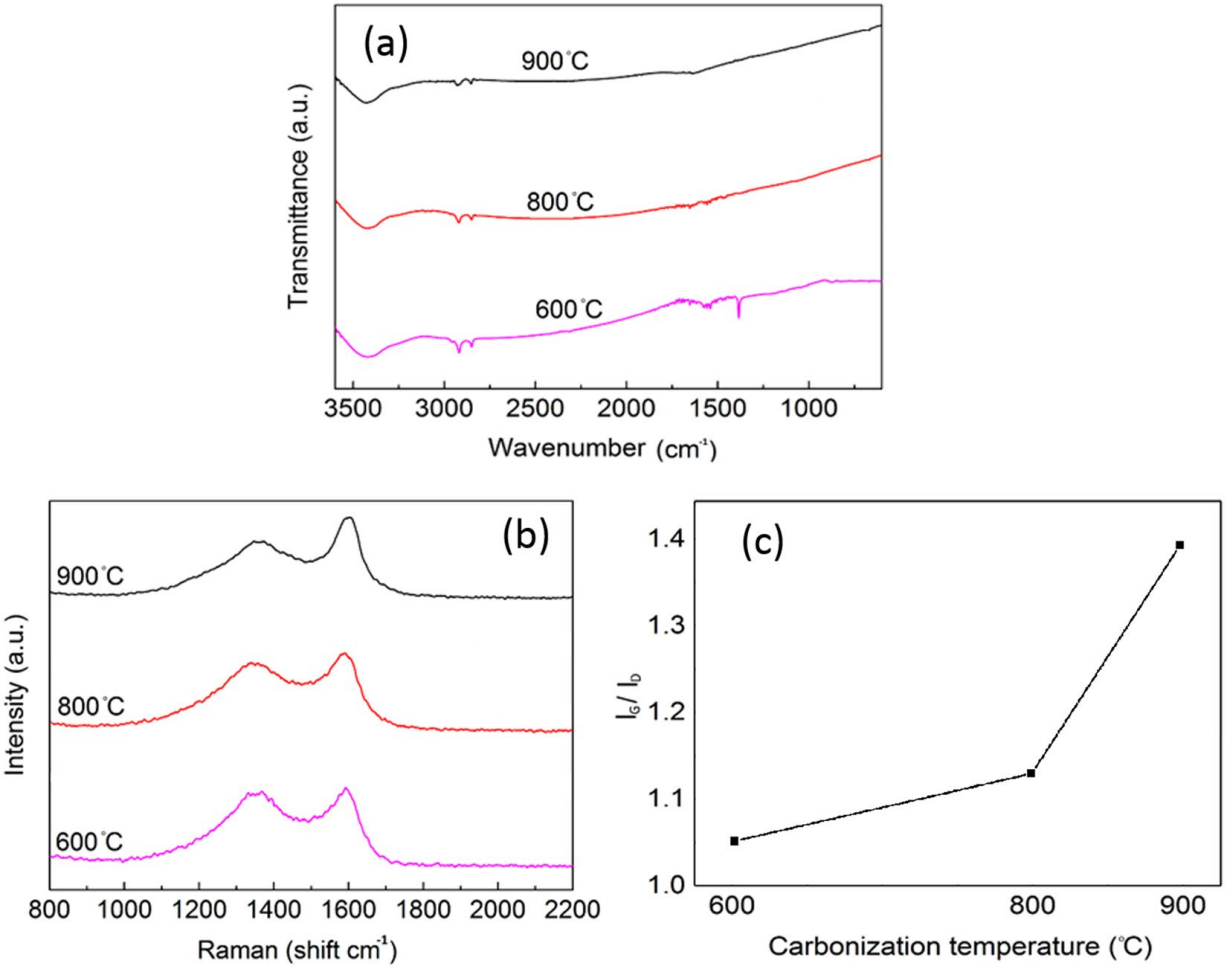
(**a**) The FTIR spectra, (**b**) Raman spectra, and (**c**) the I_G_/I_D_ ratio of samples at different carbonizing temperatures.

Figure 3a shows the FTIR spectra of carbonized ultrasonicated fibres, prepared at different temperatures. All the samples displayed a broad peak between 3100 to 3500 cm^−1^ that is attributed to stretching vibrations of hydroxyl groups^21^. The peaks at 2925 and 2850 cm^−1^ display the symmetric and asymmetric vibrations of methylene (-CH_2_-) groups in alkyl chains^22^ that were weakened with the increase of carbonization temperature. This can be caused by the decomposition of methylene bands of waxes at high temperatures. In addition, the missing peaks at 1440, 820, and 750 cm^−1^ at 800 and 900 °C are related to the decrease of carbonyl, hydroxyl, and methylene groups after carbonization. By elimination of these groups, the benzene rings get closer together, the fused-ring structure will be formed, which leads to the appearance of the hexagonal carbon networks^23,24^. The Raman spectra of sonicated carbonized cellulose at different temperatures, shown at Fig. 3b, was used to compare the crystalline structures of synthesized carbon materials at different temperatures. There are two dominant peaks of D and G, on the first-order spectrum of the carbonized samples. The D band, between 1355 and 1360 cm^−1^, corresponds to disordered structures and the G band, between 1575 cm^−1^ and 1600 cm ^−1^, shows the graphite crystallite structure^25^. The ratio of the G band (I_G_) to the D band (I_D_) intensities reveals the crystalline arrangement of carbonized cellulose at different temperatures. Similar analysis has been used to evaluate the crystalline structure of carbon materials with different heat treatment temperatures^26,27^. As seen in Fig. 3c, with the increase of carbonization temperature from 600 to 900 °C, the I_G_/I_D_ increases about 33% (from 1.05 to 1.4), suggesting rearrangement of crystalline structure to a more ordered state^28^. This rearrangement could provide an orderly charge transfer path, resulting in a higher electrical conductivity, with a faster response time^29^. Similar results were also observed in the synthesis of carbon nanotube arrays from Ferrocene, where the G/D band intensity ratio demonstrated a decline with the increase of heat treatment temperature in a certain range^26^. In this study, the sample carbonized at 900 °C was considered for further processing.

### Effect of sonication

**Figure 4:**
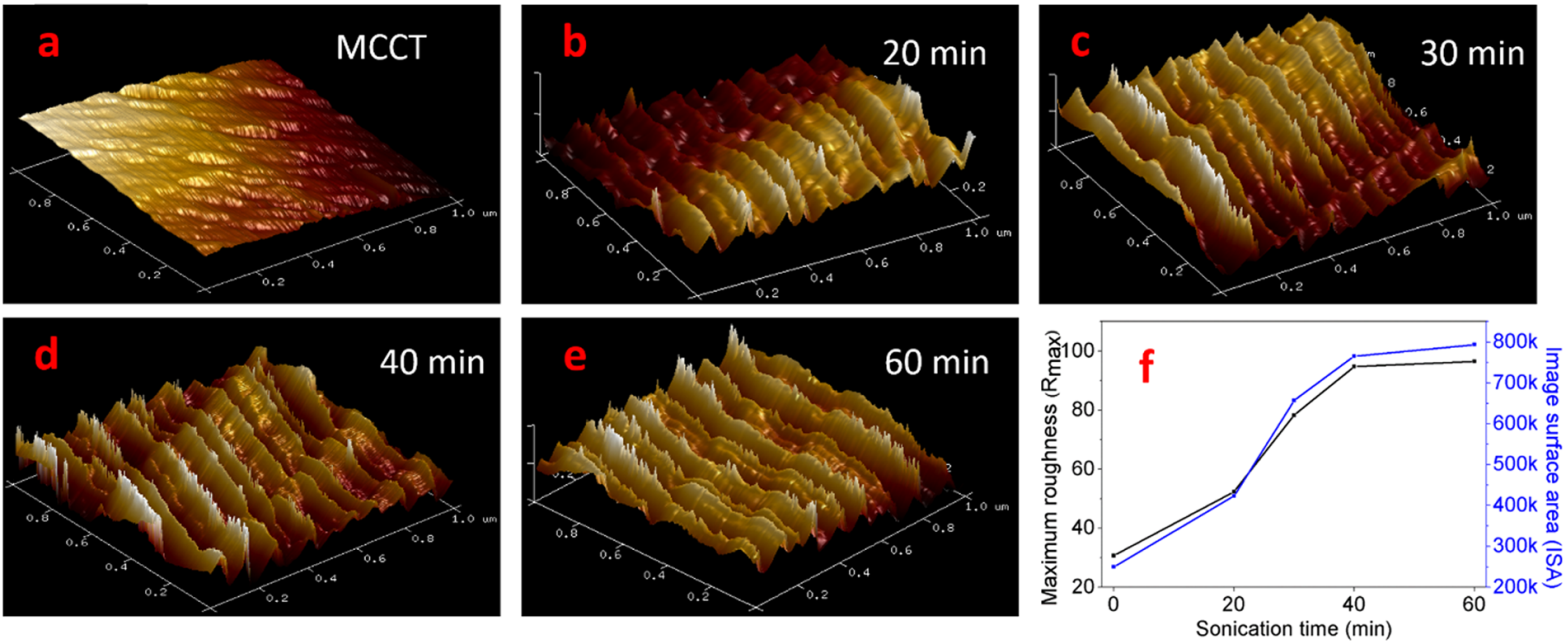
AFM surface topographies and surface relief parameters of carbonized cellulose microtubes (CCMT) and nanogrooved carbonized cellulose microtubes (NGCCMT) at different sonication time periods.

Figure 4 shows AFM images of the carbon tubes without sonication (Fig. 4a) and after being sonicated for different durations (Fig. 4b–e). In all these images the fibril bundles can be seen on the surface of carbon tubes, similar to that of cellulose fibres after chemical purification. The effect of pre-treatment can be clearly observed in the sequence of images. The nanofibrils appear to be disjointed from their bundles on the surface of carbon microtubes after sonication. For further investigation, the maximum roughness (R_max_) and the image surface area (ISA) were calculated at different times. Significant increase of R_max_ about 71%, 155%, 208%, and 214%, respectively after 20, 30, 40, and 60 minute sonication times were obtained, in comparison to that of carbonized cellulose microtubes (CCMT) (Fig. 4f). Similarly, image surface area also exhibited significant increase with the sonication time.

To investigate the effect of nanogrooving on the interactions of carbon tubes with polymer solutions, we prepared chitosan(CH)/carbon microtubes (CCMT and nanogrooved carbonized cellulose microtubes (NGCCMT) with 40-minute sonication) dispersions with the composition of 50/50 (wt%/wt%).

**Figure 5:**
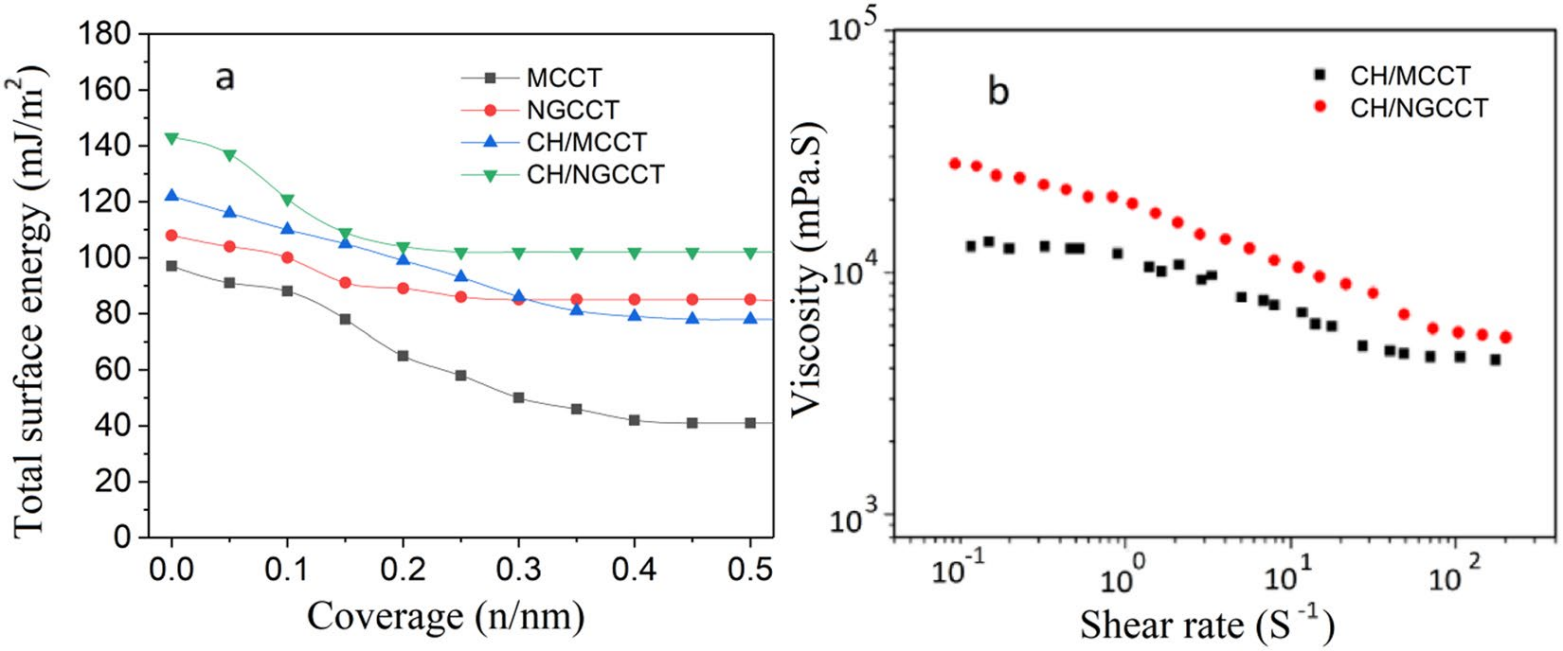
(**a**) Total surface energy profiles of CCMT, NGCCMT, CH/CCMT, and CH/NGCCMT. (**b**) Effect of nanogrooving on the viscosity of chitosan solutions.

Cohesion and physical micro interlocking of composite material components significantly depend on the surface energy parameters of matrix and filler. Here, the total surface energy (TSE) values of CCMT, NGCCMT, CH/CCMT, and CH/NGCCMT are shown in Fig. 5a. TSE is the sum of polar (chemical) and non-polar (physical) surface interactions. It was found that the nanogrooving resulted in an enhancement of the total surface energy of microtubes, indicating that the surface area and roughness have been increased. This is in good agreement with the AFM results where nanogrooving considerably increased the surface roughness parameters of carbon tubes. Surface heterogeneity explains the high percentage of the areas with a significantly different energy compared to the rest of surface and the surface energy of CCMT exhibited greater than 40% heterogeneity, whereas, NGCCMT showed about 25% heterogeneity, suggesting that the ultrasonication leads to a more uniform dispersion of energy over the entire microtube surface. Figure 5a shows the total surface energy (TSE) of chitosan/microtube composites. It is observed that the CH/NGCCMT have higher values of TSE at every surface coverage. In addition to the enhanced surface area, this phenomenon can be explained by the fact that a higher amount of the absorbed chitosan leads to increase in surface chemical activity, resulting in an enhanced polar surface interaction, compared to the CH/CCMT.

Here we also analysed the effect of nanogrooving on the rheological performance of the composite solution which is an important factor in extrusion processing. Figure 5b shows the viscosity of solutions as a function of shear rate at 25 °C. The applied torques covered a range of shear rate from 0.1 to 100 S^−1^. As shown, the flow rate of both CCMT and NGCCMT dispersions show a psuedoplastic (shear-thinning) non-Newtonian behaviour. However, chitosan/NGCCMT represented higher viscosity values in the entire shear rate range. It can be explained by the fact that the rheological behaviour of dispersions is influenced by the surface topography of the tubes^30^. The NGCCMT with larger surface areas can result in stronger physical entanglements with the polymer chains, better dispersion within the solution, and hence bigger elastic response during the test. This is in good agreement with literature results; for instance Kashiwagi^31^ reported that single wall carbon nanotubes, with finer dimensions, yield nanocomposite solutions with more gel-like rheological performance compared to that made up of multiwall carbon nanotubes.

**Figure 6:**
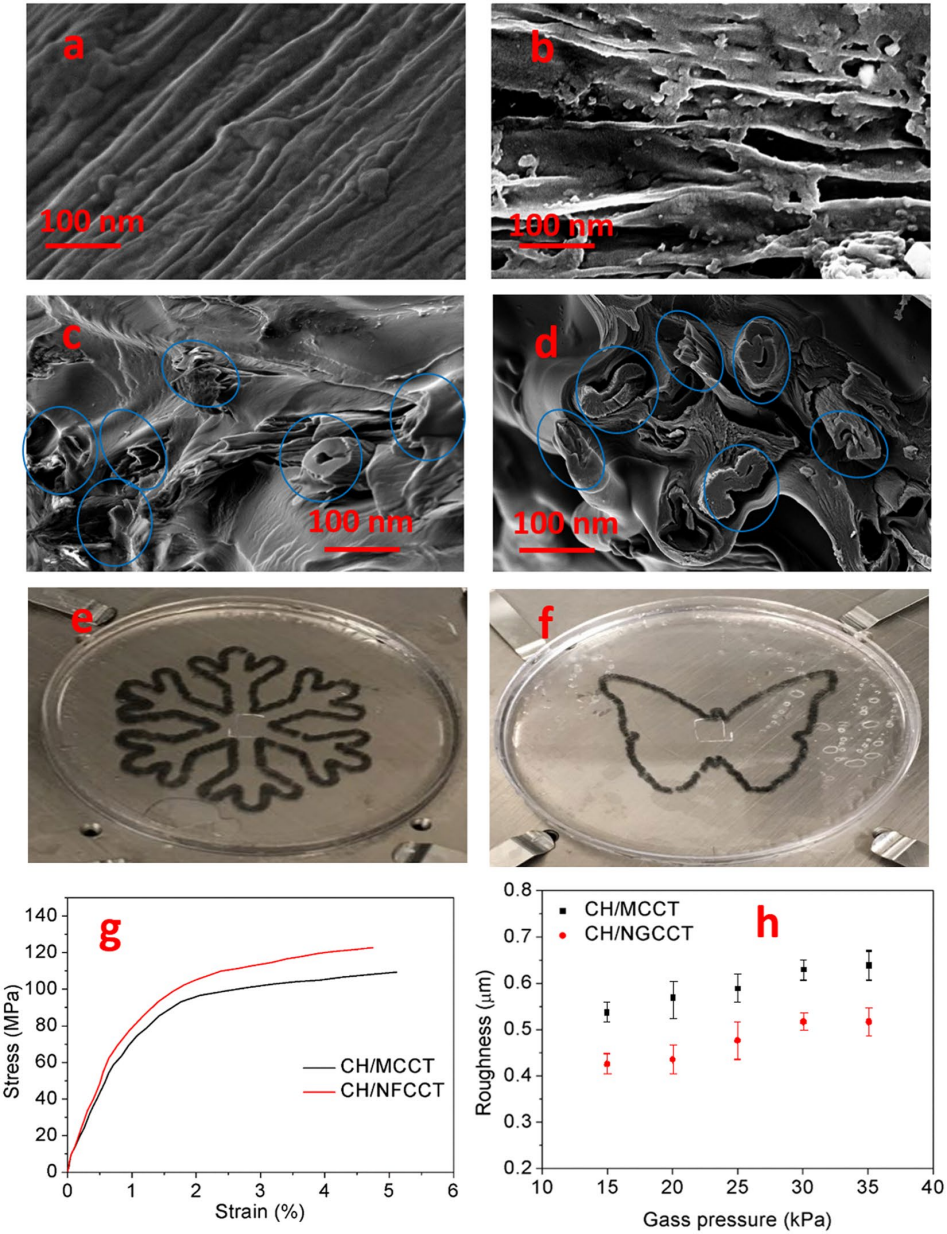
(**a**) SEM image of the longitudinal fracture surface of Chitosan/Carbonized cellulose microtubes (CH/CCMT). (**b**) SEM images of the longitudinal fracture surface of Chitosan/Nanogrooved carbonized cellulose microtubes (CH/NGCCMT). (**c**) SEM image of cross section of the printed CH/CCMT. (**d**) SEM image of the cross section of the printed CH/NGCCMT. (**e**,**f**) Demonstration of printed patterns using CH/NGCCMT. (**g**) Typical stress-strain curves of the printed fibres. (**h**) Confocal microscopy roughness measurements of the 3D printed fibres.

The dispersions were then transferred to a 3D printing syringe and spun into a 90% (v/v) cold ethanol coagulation bath through a 600 μm nozzle tip. Figure 6a,b show the SEM images of the longitudinal fracture surface of samples. The axial peeling reveals the carbon tube surfaces. Figure 6a is an image of fibre surface of the CH/CCMT, in which there are no voids between the fibrils bundles on the tube surface. Figure 6b shows the surface of the nanogrooved carbon tube made composite (CH/NGCCMT), the grooves of which become channels for the polymer matrix.

The visual verification of chitosan and carbon microtubes interaction in CH/CCMT and CH/NGCCMT composites can be seen in Fig. 6c,d. In both of the samples the fracture cross section of the composite displays an embedded network of carbon microtubes within the chitosan matrix. It can be noticed that the microtubes (blue circles), were surrounded by matrix and no filler pull out or debonding observed.

Figure 6e,f show two complex shaped CH/NGCCMT samples printed in the form of a butterfly and a snowflake. Here we studied the effect of nanogrooving on the mechanical properties of the composites. For many applications, mechanical strength of the printed materials is essential for handling and applications. Figure 6g illustrates the typical tensile behaviour of wet spun composites. The fibre made from NGCCMT has a Young’s modulus of 4.41 GPa that is almost 15% higher than that of the fibre made from CCMT (3.84 GPa). Similarly, the CH/NGCCMT fibre demonstrates a better yield strength of about 13%, in comparison to that of the CH/CCMT fibre. This difference could be due to a better shear stress transfer within CH/NGCCMT components as a consequence of increased van der Waals forces as well as higher physical entanglement between polymer matrix and the surface of nanogrooved filaments^32^. This increased radial-compressive stress transfer enhances the fracture strength under the tensile stress. Additionally, the radial-compressive stress improves with the tensile stress and therefore boosts the effectiveness of the load transfer. This is consistent with what has been reported in literature, for instance, the increase of intra-filament fracture was demonstrated using a model that predicted the radial-compressive stress in twisted filaments^33^.

However, with regard to the elongation at break of fibres, both CH/CCMT and CH/NGCCMT demonstrated almost similar performances. In the case of CH/NGCCMT, stronger matrix/filament interactions resulted in a structure with higher fracture resistance whereas CCMT might have a higher tendency for interfacial slippage of filaments due to smoother surface morphology, which manifests as higher elongation at break^34^.

The roughness measurement showed that the surface morphology of printed fibres differs based on pre-treatment of the filaments. The values were obtained with the aid of a confocal laser scanning microscope. As Fig. 6h shows, with increase of printing gas pressure from 15 to 35 KPa, the roughness of CH/CCMT fibres varied from 0.53 to 0.63 μm. Whereas, at this gas pressure range, the printed lines of CH/NGCCMT with the same composition showed a noticeable decrease, with the roughness in the range of 0.42 to 0.52 μm. This can be attributed to die swell reduction as a result of higher filler incorporation into polymer solution. The higher the physical entanglement of matrix on the surface of grooved filaments the smaller is the spherical configuration, after leaving the nozzle tip^35,36^. The importance of smooth surfaces is more pronounced in layer by layer 3D printed structures, where the rough fibres lead to unpredicted dimensions and properties as a consequence of increased interlayer defects^37^.

## Conclusion

A nanogrooved surface carbon microtube was synthesized using acoustic cavitation. It was found that the nonogrooving augmented the microtube surface energy and so improved the cohesion and physical micro interlocking interactions with the polymer matrix. The modified tubes showed increased surface area leading to decreased die swelling, as well as improved coherency and homogeneity of the extrudate. Coagulation helps achieve self-reinforcement properties where the grooved surface of carbon filaments has a synergetic effect on the entanglements, because of higher interaction with the polymer matrix. Synthesized carbon tubes with modified surface topography help prepare composite solutions with stable rheological behaviour leading to elimination of time restrictions in the extrusion process. The nanogrooved surface carbon microtube allows processing of 3D structure assemblies and offers potential for the applications where the extrudate holding time may be prolonged.

## Experimental Section

### Preparation of nanogrooved carbon tubes

First, the cotton linters were washed with distilled water and dried. The dried fibres were soaked in dilute alkaline aqueous solution (5 wt%) and the mixture was put in an autoclave at 150 °C for 2 h, followed by washing with distilled water until it became neutral. To remove the ashes, cotton was first bleached with sodium hypochlorite (NaClO, chlorine content/cotton linter: 1.5/100 (wt/wt)) and washed with distilled water. Then, the fibres were hydrolysed with dilute hydrochloric acid (acid/cotton linter: 2/100(wt/wt)), neutralized with ample distilled water, and dried. After chemical purification, the fibres were soaked in distilled water with a concentration of 1 wt%. Then, 125 ml of the dispersion was placed in a high intensity ultrasound generator (UIP1000hdT). The process was conducted at an output power of 400 W, with a frequency of 20 kHz, for 40 minute. To control temperature, the treatment was carried out in an ice bath. Then, the purified cotton fibres were carbonized in a clean room furnace (Furnace Ceramic Tube Tetlow), under nitrogen, with a heating rate of 10 °C/min. The temperature for carbonization was controlled at 600, 700, 800, and 900 °C, for one hour.

### Extruding/coagulating

The extrusion solution is comprised of chitosan. Medium molecular weight chitosan with a deacetylation degree of 75–85% was purchased from Sigma Aldrich (Sydney, Australia) and used without further purification. Chitosan was found to be selective in the noncovalent wrapping of carbon tubes and specially disperse them over other impurities^8^. The solution was prepared by dissolving of 3 g chitosan (Medium molecular weight chitosan with deacetylation degree of 75–85% (Sigma Aldrich)) in 100 ml acetic acid (1% v/v). The solution was stirred at room temperature for 6 h. Then, carbon tubes, either untreated or ultrasonicated, were dispersed in chitosan solution at a concentration of 50 wt%, per chitosan weight, with the aid of sonication in a low power ultrasonic bath. The dispersion was stored into a 5 ml syringe that was fixed on a steel micronozzle (0.6 mm in diameter). The extrusion process was performed using a bioplotter 3D printer on a 3 axis stage, where the motion was controlled using a preprogramed patterning procedure. The printing flow was controlled by the air pressure. The ink was printed (with the nozzle feed rate of 5 mm/s) and then allowed to coagulate in a non-solvent bath. The height of liquid in the coagulation bath was 1 cm above the printing materials. Finally, the printed materials were dried in air.

### Characterization

To evaluate the effect of chemical purification on the weight loss behaviour of the cotton fibres, thermal gravimetric analysis was applied out by means of a TG analyser (Rheometric scientific TGA), with the heating rate of 10 °C/min.

Scanning electron microscopy (SEM) was carried out to analyse the quality and dimension of fibres using a Zeiss instrument (Zeiss Supra 55VP).

Atomic force microscopy (AFM) was used to evaluate the effect of ultrasonication on the surface topography of carbon microtubes (Bruker Multimode 8).

The surface energy of the samples were measured by an Alperton machine (surface energy analyser, middlesex, UK). For each test, 1.0 g of microtube or chitosan/microtube composite was packed into the a silanized glass column. As a test preparation, before each measurement the sample was conditioned at 30 °C and under helium carrier gas for 2 h.

To evaluate the viscosity behaviour, the dispersions were tested by a stress controlled rheometer. They were stirred for 3 h at 80 °C, cooled to room temperature and analysed in a shearing time of 15 S.

Tensile properties of the printed materials were tested according to ASTM D 3822 by an Instron machine (model 5567, USA).
